# Psychological and Behavioral Response to the Coronavirus (COVID-19) Pandemic

**DOI:** 10.7759/cureus.7923

**Published:** 2020-05-02

**Authors:** Fizra Balkhi, Aamna Nasir, Arhama Zehra, Ramsha Riaz

**Affiliations:** 1 Internal Medicine, Jinnah Sindh Medical University, Karachi, PAK; 2 Medicine, Dow Medical College, Dow University of Health Sciences, Karachi, PAK; 3 Internal Medicine, Dow Medical College, Dow University of Health Sciences, Karachi, PAK

**Keywords:** pandemic, corona virus, covid-19, behavioral impact, psychological impact, karachi, pakistan

## Abstract

Background

The outbreak of Coronavirus (COVID-19) in Wuhan, China, which began in December 2019, evolved to become a global pandemic. The pandemic, along with the obvious health-related impact, also poses a serious threat to the psychological well-being of individuals and has resulted in significant behavioral changes. We aimed to describe the psycho-behavioral response to this crisis among the population of Karachi, Pakistan, in the month of March 2020.

Methods

A structured, self-administered questionnaire was constructed, based on previously conducted surveys, assessing the psychological impact and behavioral changes pertaining to COVID-19. Questionnaires were made available online, and were administered to any individual who was a resident of Karachi, during March 2020. Data were analyzed using Statistical Package for Social Sciences (SPSS) version 21.0 (IBM Corp., Armonk, NY) to identify possible risk factors for psychological and behavioral changes. The responses were compared based on gender, age, and level of education, to find possible statistical correlations using chi-square test.

Results

This research studied data from 400 participants residing in Karachi, Pakistan. The spread of the virus had resulted in subsequent development of fears in the target population, with the majority of the respondents feeling anxious on a daily basis (62.5%). The participants feared going to marketplaces (88.8%), were concerned for the health of their family members (94.5%), and felt under-confident with the current infection control measures (71%). Significantly elevated levels of fear were noted among people >35 years of age. They were more likely to fear for the safety of their health even at home (p=0.06). Meanwhile, increased levels of anxiety due to use of social media among people below 35 years had resulted in avoidance behaviors (p=0.04). There was a higher tendency for graduates to fear for the safety of their health, even at home (p<0.01). In addition, more than three-fourths of our participants had incorporated changes in their behavior to ensure their safety i.e. reduced physical contact (86.5%) and visits to healthcare facilities (74.5%), canceled plans (84.5%), and washing hands more often (87%).

Conclusion

Our study highlighted the increased anxiety levels that an individual experienced on a regular basis regarding their health, the health of their peers, certain avoidance behaviors as a result of the disease, and behavioral changes of the concerned population. Besides calling attention to this worrisome situation, we also tried to list possible solutions to avert any future distress that may ensue as a result. Hopefully, our study will help the concerned authorities to take measures in order to alleviate the psychological and behavioral impact of COVID-19.

## Introduction

Coronavirus disease (COVID-19), a new strain belonging to the family of coronaviruses which includes the Severe Acute Respiratory Syndrome (SARS)-CoV and the Middle East Respiratory Syndrome (MERS)-CoV, was first isolated in January 2020 by Chinese scientists, in Wuhan, Hubei province, China [1-2]. The outbreak hit Wuhan in late December 2019, when a large number of patients presented with pneumonia of unknown etiology [1]. This novel virus targets the respiratory system and the symptoms range from mild clinical manifestations such as dry cough, shortness of breath, sore throat, and fever to numerous fatal complications including severe bilateral pneumonia, Acute Respiratory Distress Syndrome (ARDS), septic shock, and eventually multi-organ failure [3-4]. The pandemic, as declared by the World Health Organization (WHO) on March 11, 2020, has caused havoc globally, hitting 169 countries, and affecting every continent except Antarctica. Till April 14, 2020, the number of confirmed cases had gone over 1 844 863, and 117 021 deaths had been reported worldwide [5-6].

In order to end a pandemic, population co-operation is extremely vital. The WHO has stated that it is necessary to communicate the risk and engage with different communities to come up with an effective program in order to protect and prepare ourselves against COVID-19, as many studies have shown that practicing preventive measures on an individual level is effective in curbing the spread of infection [7-8]. The WHO has also advised the general public to follow some basic precautionary measures, i.e. wearing a mask, washing hands, using a hand sanitizer, maintaining social distancing, and staying at home, all of which are now strictly enforced by the government of Pakistan [9]. As of now, limited data is available exploring to what extent these recommendations are being followed.

As the current focus of most of the researches revolves around the pathophysiology, clinical manifestation, diagnosis, and treatment of the disease, the mental health aspect of the outbreak is often overlooked and little to no research coverage is given to understanding the psychological impact and behavioral changes in the affected population [1,10-12]. Moreover, standard data collection and analysis in controlling an outbreak seldom include public perception, beliefs, attitudes, and practices towards the overwhelming situation. Considering that the current situation has taken a heavy toll on most peoples’ mental health and addressing the situation is the need of the hour, the WHO released some mental health considerations that should be followed during this crisis. Some of these are to avoid watching and listening to news constantly, staying connected with loved ones through digital media, reassuring and supporting each other, along with taking care of one’s own health i.e. exercising, eating healthy, and sleeping well regularly [13].

With this research, we aim to address the psycho-behavioral changes in the population of Karachi, Pakistan after the COVID-19 outbreak in terms of psychological distress i.e. anxiety and fears, along with incorporation of different behavioral practices i.e. rate of avoidance behavior and implementation of non-pharmacological preventive measures, while discussing possible solutions to deal with these changes more effectively. Furthermore, it is significant to state that until now no such study focusing on the psychological and behavioral impact of the pandemic has been conducted in the region.

## Materials and methods

We carried out a descriptive, cross-sectional study in the month of March 2020 in Karachi, Pakistan. The sample size was calculated using the OpenEpi sample size calculator [14]. With a confidence level of 95%, the estimated sample size was 375 using an anticipated frequency (p) of 57.7% [15]. We selected and surveyed 430 candidates for statistical convenience, out of which 30 responses were discarded. Study participants were selected using a convenience sampling technique and sampling was done by sending an online questionnaire due to a lockdown all over Karachi. Our study participants included all those who were residents of Karachi during this pandemic and no discrimination was made on the basis of age, gender, or ethnicity. Only those who were not residents of Karachi were excluded from this survey.

A structured, self-administered questionnaire was constructed unanimously by the authors on the basis of previously done surveys with regards to Middle East Respiratory Syndrome (MERS) [8, 16]. The questionnaire was made available online via Google forms. Before conducting the actual survey, a pilot study comprising 10 participants was carried out to ensure clarity of the questionnaire. A consent form was attached before our questionnaire giving us permission to use the collected data. Confidentiality and anonymity were thoroughly ensured and no names or email addresses were asked. The data was collected over a period of five days from March 19, 2020 to March 24, 2020.

The questionnaire consisted of 24 questions, focusing on the psychological impact and behavioral changes of the participants pertaining to the pandemic. In the first section of the questionnaire, individual characteristics were asked which included age, gender, and the level of education. The rest of the questionnaire was divided into two parts. Concerns about the disease, its severity, personal efforts, and satisfaction regarding governmental efforts to fight the disease were evaluated using 21 questions. Each question had a ‘Yes’ or ‘No’ response. The first part focused on assessing the psychological impact of COVID-19. It included questions regarding fear; whether the participant experienced fear while leaving their house, visiting a crowded place, or when a family member went outside the house. The questions also included if they feared for their health or their family members’ health even when at home. The respondents were further asked if they felt anxious on a daily basis because of COVID-19 and whether they were confident with the current infection control measures. The questions also dealt with their concerns regarding the isolation of patients by the government. Participants were asked if they perceived fake news over social media as a possible reason for panic among the masses or how grave they believed the current situation was. The second part of the questionnaire focused on assessing the behavioral changes among the participants; whether they had limited their physical contact, avoided healthcare facilities/prayer places, or canceled any plans out of fear of COVID-19. The final questions of the questionnaire dealt with the participant’s hygienic practices which included hand washing, use of a hand sanitizer, and use of a mask.

The data was analyzed using Statistical Package for the Social Sciences software (SPSS version 21.0; IBM Corporation, Armonk, NY, USA). Chi-squared test was applied to compare responses based on gender, age, and the level of education, to find possible statistical correlations. A p-value of <0.05 was considered statistically significant.

## Results

Personal characteristics

A total of 400 residents of Karachi participated in this study, out of which 74% (n=296) were younger than 35 years, whereas the remaining 26% (n=104) were above 35 years of age. An equal number (n=200, 50%) of males and females participated in our study, of which 45% (n=180) were undergraduates and the rest were graduates (n=220, 65%). These demographics are listed in Table 1.

**Table 1:** Demographics of the study participants

Characteristics	Variables	N (%)
Gender	Male	200 (50)
	Female	200 (50)
Age	Less than 35 years	296 (74)
	More than 35 years	104 (26)
Level of education	Undergraduate	180 (45)
	Graduate	220 (65)

Psychological impact of COVID-19

The first section of our questionnaire which explored the psychological impact of the ongoing pandemic, underlined some interesting results, as outlined in Table 2. While two-thirds (64.3%) of our participants were apprehensive of leaving their homes because of the corona virus, comparatively more (83.8%) felt fearful if a family member went outside. A large fraction of our participants (88.8%) feared visiting crowded places such as markets and departmental stores, and felt safer inside their homes (58%). Almost all of our participants (95.3%) unanimously agreed that the government should isolate the infected patients in separate hospitals while also expressing their (71%) under-confidence in the current infection control measures. It is concerning that the ongoing pandemic has made two-thirds (62.5%) of our participants feel anxious on a daily basis. A huge majority (82.8%) deemed fake news surfacing on the social media as a possible reason for the panic which ensued after the outbreak. While around three-fourths (71.5%) of the respondents realized the gravity of the situation, the remaining (28.5%) still believed the situation was not as bad as it was being portrayed.

**Table 2:** Psychological impact of COVID-19

Statements	Yes	No
I fear leaving my house because of COVID-19.	257 (64.3%)	143 (35.8%)
I fear visiting crowded places i.e. markets and departmental stores.	355 (88.8%)	45 (11.3%)
I fear for the safety of my health even when I'm at home.	168 (42.0%)	232 (58.0%)
I fear for the health of my family members.	378 (94.5%)	22 (5.5%)
I feel anxious when a family member goes outside the house.	335 (83.8%)	65 (16.3%)
I feel anxious on a daily basis because of COVID-19.	250 (62.5%)	150 (37.5%)
I feel that the government should isolate COVID-19 patients to specific hospitals.	381 (95.3%)	19 (4.8%)
I feel under-confident with the current infection control measures.	284 (71%)	116 (29.0%)
I feel fake news surfacing through social media regarding COVID-19 is causing panic.	331 (82.8%)	69 (17.3%)
I feel the situation is not as bad as it is being portrayed.	114 (28.5%)	286 (71.5%)

Behavioral impact of COVID-19

Our second section of the questionnaire dealt with the behavioral impact of COVID-19, as demonstrated in Table 3. Only a small fraction of our participants had pretended to be sick (14.8%) to avoid going to their workplace/educational institute or considered quitting or applying for a leave (24.8%) because of COVID-19. It is reassuring that more than three-fourths of our participants had incorporated changes in their behavior to ensure their safety. In addition, more than three-fourths participants had limited their physical contact with people (86.5%), avoided/reduced their visits to healthcare facilities (74.5%), had recently cancelled their plans such as family reunions, social gatherings, traveling or meetings (84.5%) and washed their hands more frequently (87%). However, fewer participants had reduced/avoided going to prayer places (63.5%), carried a hand sanitizer with them (56.8%), or wore a mask (44.3%). Since watching/listening/reading the current news increased anxiety levels, around one-third (35%) of our participants had started to avoid it. Notably, half of our participants (54.5%) had purchased groceries out of fear of them running out.

**Table 3:** Behavioral impact of COVID-19

Statements	Yes	No
I have thought of quitting or applying for leave at the workplace/educational institute because of COVID-19.	99 (24.8)	301 (75.2%)
I have pretended to be sick to avoid going to the workplace/educational institute.	59 (14.8%)	341 (85.2%)
I have limited my physical contact with people.	346 (86.5%)	54 (13.5%)
I have recently avoided/ reduced using healthcare facilities.	298 (74.5%)	102 (25.5%)
I have recently avoided/ reduced going to prayer places.	254 (63.5%)	146 (36.5%)
I have recently started to avoid watching, reading or listening to news because it made me anxious.	140 (35.0%)	260 (65.0%)
I have recently cancelled my plans i.e. family reunions, social gatherings, travelling or meetings because of COVID-19.	338 (84.5%)	62 (15.5%)
I have recently purchased groceries out of fear of them running out.	218 (54.5%)	182 (45.5%)
I wash my hands more frequently.	348 (87%)	52 (13.0%)
I carry a hand sanitizer all the time.	227 (56.8%)	173 (43.3%)
I have started wearing a mask because of COVID-19.	177 (44.3%)	223 (55.8%)

Comparison between different groups

Chi-square tests were conducted and the results of the psychological and behavioral impact of COVID-19 were compared between different demographic variables such as gender (males/females), age groups (less than 35 years/more than 35 years), and level of education (graduates/postgraduates), as shown in Table 4.

**Table 4:** Assessment of the psychological and behavioral impact of COVID-19 according to various demographic variables ^a^Calculated using Chi-square test; P-value <0.05 considered statistically significant

Statements	Males	Females	P-value^a^	<35 years	>35 years	P-value^a^	Undergraduate	Graduate	P-value^a^
I fear leaving my house because of COVID-19	115 (57.5%)	142 (42.5%)	<0.01	195 (65.8)	62 (59.6%)	0.25	120 (66.6%)	137 (62.2%)	0.36
I fear visiting crowded places i.e. markets and departmental stores	176 (88%)	179 (89.5%)	0.64	264 (89%)	91 (87.5%)	0.64	163 (90.5%)	192 (87.2%)	0.30
I fear for the safety of my health even when I'm at home	88 (44%)	80 (40%)	0.42	116 (39%)	52 (50%)	0.06	61 ((33.8%)	107 (48.6%)	<0.01
I fear for the health of my family members	189 (94.5%)	189 (94.5%)	1.00	283 (95.6%)	95 (91.3%)	0.10	173 (96.1%)	205 (93.1%)	0.20
I feel anxious when a family member goes outside the house	163 (81.5%)	172 (86%)	0.22	244 (82.4%)	91 (87.5%)	0.23	144 (80%)	191 (86.8%)	0.07
I feel anxious on a daily basis because of COVID-19	132 (66%)	118 (59%)	0.15	174 (58.7%)	76 (73%)	0.01	90 (50%)	160 (72.7%)	<0.01
I feel that the government should isolate COVID-19 patients to specific hospitals	192 (96%)	189 (94.5%)	0.48	284 (95.9%)	97 (93.26%)	0.27	173 (96.1%)	208 (94.5%)	0.46
I feel under-confident with the current infection control measures	136 (68%)	148 (74%)	0.19	210 (70.9%)	74 (71.1%)	0.97	131 (72.7%)	153 (69.5%)	0.48
I feel fake news surfacing through social media regarding COVID-19 is causing panic	167 (83.5%)	164 (54.6%)	0.70	244 (82.4%)	87 (83.6%)	0.78	151 (83.8%)	180 (81.8%)	0.59
I feel the situation is not as bad as it is being portrayed	62 (31%)	52 (26%)	0.27	80 (27%)	34 (32.6%)	0.27	50 (27.7%)	64 (29%)	0.77
I have thought of quitting or applying for leave at the workplace/educational institute because of COVID-19.	57 (28.5%)	42 (21%)	<0.01	72 (24.3%)	27 (25.9%)	0.11	36 (20%)	63 (28.6%)	0.03
I have pretended to be sick to avoid going to the workplace/educational institute.	32 (16.0%)	27 (13.5%)	<0.01	48 (16.2%)	11 (10.5%)	<0.01	25 (13.8%)	34 (15.4%)	0.67
I have limited my physical contact with people	174 (87%)	172 (86%)	0.77	258 (87%)	88 (84.6%)	0.51	154 (85.5%)	192 (87.2%)	0.62
I have recently avoided/ reduced using healthcare facilities.	142 (71%)	156 (78%)	0.11	220 (74.3%)	78 (75%)	0.89	143 (79.4%)	155 (70.4%)	0.04
I have recently avoided/ reduced going to prayer places.	118 (59%)	136 (68%)	0.06	190 (64%)	64 (61.5%)	0.63	122 (67.7%)	132 (60%)	0.11
I have recently started to avoid watching, reading or listening to news because it made me anxious.	63 (31.5%)	77 (38.5%)	0.14	112 (37.8%)	28 (26.9%)	0.04	62 (34.4%)	78 (35.4%)	0.83
I have recently cancelled my plans i.e. family reunions, social gatherings, travelling or meetings because of COVID-19.	171 (85.5%)	167 (83.5%)	0.58	249 (84%)	89 (85.5%)	0.72	151 (83.8%)	187 (85%)	0.76
I have recently purchased groceries out of fear of them running out.	99 (49.5%)	119 (59.5%)	0.04	165 (55.7%)	53 (50.9%)	0.40	107 (59.4%)	111 (50.4%)	0.07
I wash my hands more frequently	182 (91%)	166 (83%)	0.02	257 (86.8%)	91 (87.5%)	0.86	151 (83.8)	197 (89.5%)	0.09
I carry a hand sanitizer all the time.	110 (55%)	117 (58.5%)	0.48	175 (59.1%)	52 (50%)	0.11	104 (57.7%)	123 (55.9%)	0.71
I have started wearing a mask because of COVID-19.	84 (42.0%)	93 (46.5%)	0.36	132 (44.5%)	45 (43%)	0.82	83 (46.1%)	94 (42.7%)	0.50

A comparison between the genders did not yield any significant differences except in a few domains. Males were more likely to fear leaving their homes after the pandemic (p<0.01), had pretended to be sick (p<0.01) to avoid going to their workplace/educational institute, and considered quitting or applying for a leave (p<0.01) because of COVID-19. On the other hand, females were more likely to purchase additional amounts of groceries in fear of them running out (P=0.04). In our study, we discovered that the number of times males washed their hands after the outbreak was more than females (p=0.02).

Our research highlighted that there was a higher tendency of people above the age of 35 years to feel anxious on a daily basis after the outbreak (p=0.01). Meanwhile, the younger age group, below 35 years of age, felt anxious as a result of watching/reading or listening to the news and hence has started to avoid it (p=0.04). They were also more likely to have pretended to be sick in order to avoid going to the workplace/educational institute (p<0.01).

While drawing out a comparison between graduates and undergraduates, our study highlighted the higher tendency of graduates to fear for the safety of their health even at home (p<0.01) and to feel anxious on a daily basis after the outbreak (p<0.01). However, a higher number of undergraduates had thought of quitting or applying for leave of absence at the workplace/educational institute because of COVID-19 (p=0.03) or had recently avoided/reduced using healthcare facilities (p=0.04).

## Discussion

As COVID-19 continues to spread across the world, the importance of public cooperation in ensuring preventative measures to halt the spread of the disease has increased. A number of researches on the previous outbreak of SARS highlighted the importance of psycho-behavioral impact of an outbreak on the population [17-18]. However, an extensive literature search yielded no significant survey conducted to assess the psychological and behavioral impact of the coronavirus outbreak in the country of Pakistan. Therefore, it is apt to state that our research aimed to fill in this gap by assessing the possible psychological and behavioral repercussions of this pandemic, while devising possible solutions to avert any future distress that may ensue as a result.

Overall, our study yielded the majority of respondents (62.5%) stating that they feel anxious due to the ongoing coronavirus, which is a very alarming situation. A study conducted during the initial stages of COVID-19 in China concluded that about 28.8% of their respondents claimed to have moderate to severe anxiety symptoms [19]. A few months into the pandemic, this rising trend signifies how important it is to control the mental impact on the population in order to alleviate their anxiety levels.

Interestingly, while two-thirds of our participants were apprehensive of leaving their house, a higher fraction (83.8%) felt anxious, if it was their family member who had left instead. This might point out that an additional worry for the welfare of family members in times of crisis like these further plays a role in heightening fears. Following the start of the outbreak from a marketplace in Wuhan and the constant campaigning by the concerned authorities to avoid crowded places was probably the reason why a large fraction of our respondents (88.8%) feared visiting crowded places. This is significant because previously an association between anxiety levels and avoidance behavior including traveling or visiting public places has been established [15].

The internet (93.5%) was the primary source of health information for the general public during the initial stage of the COVID-19 outbreak in China [19]. However, it is alarming that 82.8% of our targeted population considered social media as a cause of increasing panic. We found out that 71% of our respondents also expressed concerns over the current infectious control measures; thus, supporting a study that indicated that increased levels of anxiety may lead to mistrust and dissatisfaction in the measures being taken by the government [20]. Similarly, the increasing distrust in governmental measures deduced in this research may be the reason why people are stocking up on groceries.

A previous study focusing on the SARS epidemic in 2003 found that fewer people avoided visiting hospitals [21]. This finding is contrary to our research which established that higher rates of people (74.5%) had avoided health care facilities. This could possibly be due to increased awareness in the public of Karachi, which is very encouraging because rapid nosocomial spread of diseases have been reported previously, such as the SARS outbreak which accounted for 49.3% of cases [22].

It was relieving to find that more than 80% of our participants had limited their contact with people, and canceled their plans (84.5%) which could have aided contact transmission, and incorporated a hand hygiene regime. These high levels of awareness and positive behavioral changes could be because this research was conducted at a time when preventive measures were being highly emphasized to prevent the spread of the disease. However, one should not forget that abrupt changes in lifestyle and social interaction could further trigger anxiety, especially keeping in view the uncertainty of the situation. Supporting this, a survey indicates that preventive measures are undoubtedly closely related to the effective and timely transmission of epidemic and virus-related information [20].

As males are mostly the breadwinners of the family in Pakistani households, they were predictably more perturbed about leaving their house and even considered requesting a leave of absence and were more likely to feel anxious, which negates a previous study carried out in Saudi Arabia [23]. Additionally, our study also highlighted that other than males, graduates, and people above the age of 35 years who collectively make up the majority of the workforce in a Pakistani households, also felt anxious on a day to day basis because of COVID-19. This might be due to heightened fears as a result of estimated global economic shutdown and subsequent recession of the economy [24].

In light of the patterns found in this research and previous studies, there are a number of measures that may be taken to counteract this outbreak in an efficient way. Firstly, there is a dire need to prevent the spread of fallacious information over social media and form portals where people can obtain information from reputable government sources. Secondly, the government should focus on making their future endeavors clearer and provide updated information to both the general public and health care providers in order to alleviate their anxiety levels and build their trust in the government to ensure future cooperation. Training should be provided to undergraduate medical students so they can provide counseling and therapy because as chances of contact transmission and the number of cases increase, medical help sought from medical students will become crucial [24].

Even though only a third of our participants continued to visit prayer places (36.5%), it still calls for the attention of spiritual leaders in the society to play their part and clear the misconceptions of the public, to explain to them that visiting such places in the event of an infectious outbreak would only lead to a faster spread of the disease, meanwhile also providing them with alternate ways of continuing their religious activities. In order to avoid causing huge losses to firms, work from home should be encouraged in every workplace where possible, and since young people are more receptive towards smartphone applications, students should be provided with online courses and lectures which may help the country save itself from any long-term losses [25].

Lastly, as has been noted from previous pandemics, increased anxiety leads to further exacerbation of the disease, therefore a few measures could be taken on an individual level to reduce this anxiety and fear [26]. Avoiding excessive exposure to news that would lead to distress may be helpful. Similarly, maintaining a healthy lifestyle will not only increase immunity but also help keep the mood elevated. Talking to friends and family is yet another way to gain emotional support and keep oneself comfortable and consolidated [27].

Our study naturally faced some limitations worth mentioning. Firstly, the timings of the survey greatly influenced the responses collected which may have varied if the survey was conducted at early or later stages of the pandemic. Secondly, because of an online survey, bias could not be eliminated and language barriers could not be bridged. In addition, any pre-existing psychiatric conditions among the participants were also not considered. The study also mostly focused on the urban population and the responses in rural areas might have significantly differed. 

## Conclusions

As the coronavirus continues to spread, more efforts are being invested in preventing its spread, treating the infected individuals, and developing vaccines. Amidst this, little attention is given on the psychological and behavioral impact of this disease which was evident by the lack of studies in this regard. Our study highlighted the increased anxiety levels that an individual experienced on a routine basis regarding their health and the health of their peers, certain avoidance behaviors which the disease had led to, and behavioral changes of the concerned population. Other than calling attention to this worrisome situation, we also tried to list possible solutions to avert any future distress that may ensue as a result. Hopefully, our study will help the concerned authorities to take measures in order to alleviate the psycho-behavioral impact of COVID-19. Furthermore, as the disease continues to evolve, future, larger-scale studies should be conducted to assess the psycho-behavioral impact of COVID-19 on the wider population.

## Appendices

Questionnaire:

Gender

a) Male

b) Female

Age: 

a) Less than 35 years old

b) More than 35 years old

Level of education: 

a) Undergraduate

b) Graduate

I fear leaving my house because of COVID-19.

a) Yes

b) No

I fear visiting crowded places i.e. markets and departmental stores.

a) Yes

b) No

I fear for the safety of my health even when I'm at home.

a) Yes

b) No

I fear for the health of my family members.

a) Yes

b) No

I feel anxious when a family member goes outside the house.

a) Yes

b) No

I feel anxious on a daily basis because of COVID-19.

a) Yes

b) No

I feel that the government should isolate COVID-19 patients to specific hospitals.

a) Yes

b) No

I feel under-confident with the current infection control measures.

a) Yes

b) No

I feel fake news surfacing the social media regarding COVID-19 is causing panic .

a) Yes

b) No

I feel the situation is not as bad as it is being portrayed.

a) Yes

b) No

I have thought of quitting or applying for leave at the workplace/educational institute because of COVID-19.

a) Yes

b) No

c) Does not apply to me

I have pretended to be sick to avoid going to the workplace/educational institute.

a) Yes

b) No

c) Does not apply to me

I have limited my physical contact with people.

a) Yes

b) No

I have recently avoided/ reduced using healthcare facilities (hospitals/public health care centers).

a) Yes

b) No

I have recently avoided/ reduced going to prayer places (mosque/temple/church etc).

a) Yes

b) No

I have recently started to avoid watching, reading or listening to news because it made me anxious.

a) Yes

b) No

I have recently cancelled my plans i.e. family reunions, social gatherings, travelling or meetings because of COVID-19.

a) Yes

b) No

I have recently purchased groceries out of fear of them running out.

a) Yes

b) No

I wash my hands more frequently

a) Yes

b) No

I carry a hand sanitizer all the time.

a) Yes

b) No

I have started wearing a mask because of COVID-19

a) Yes

b) No
